# Multiplex influences on vigilance and biochemical variables induced by sleep deprivation

**DOI:** 10.3389/fspor.2024.1412044

**Published:** 2024-06-28

**Authors:** Shiqi Liu, Xiaohong Ma, Ying Chen, Yuanyuan Zhao, Rujia Luo, Zhouying Wu, Yicheng Li, Yongyu Qian, Wenwen Wang, Shuohan Dong, Zengxuan Zhou, Silin Li, Yi Xiao, Xinhai Zhu, Yu Tian, Jinhu Guo

**Affiliations:** ^1^ School of Life Sciences, Ministry of Education (MOE) Key Laboratory of Gene Function and Regulation, State Key Laboratory of Biocontrol, Sun Yat-sen University, Guangzhou, China; ^2^ Engineering Research Center of Human Circadian Rhythm and Sleep, Space Science and Technology Institute, Shenzhen, China; ^3^ National Key Laboratory of Human Factors Engineering, China Astronaut Research and Training Center, Beijing, China; ^4^ Sun Yat-sen University Instrumental Analysis & Research Center, Sun Yat-sen University, Guangzhou, China

**Keywords:** sleep deprivation, circRNA, inflammation, neurotransmitter, circadian rhythm

## Abstract

**Introduction:**

Sleep loss and sleep deprivation (SD) cause deleterious influences on health, cognition, mood and behaviour. Nevertheless, insufficient sleep and SD are prevalent across many industries and occur in various emergencies. The deleterious consequences of SD have yet to be fully elucidated. This study aimed to assess the extensive influences of SD on physiology, vigilance, and plasma biochemical variables.

**Methods:**

Seventeen volunteers were recruited to participate in a 32.5-h SD experiment. Multiple physiological and cognitive variables, including tympanic temperature, blood oxygen saturation (SaO_2_), and vigilance were recorded. Urinal/salivary samples were collected and subjected to cortisol or cortisone analysis, and plasma samples were subjected to transcriptomic analysis of circular RNA (circRNA) expression using microarray. Plasma neurotransmitters were measured by targeted metabolic analysis, and the levels of inflammatory factors were assessed by antibody microarray.

**Results:**

The volunteers showed significantly increased sleepiness and decreased vigilance during SD, and the changes in circadian rhythm and plasma biochemistry were observed. The plasma calcium (*p *= 0.0007) was induced by SD, while ischaemia-modified albumin (IMA, *p *= 0.0030) and total bile acid (TBA, *p *= 0.0157) decreased. Differentially expressed circRNAs in plasma were identified, which are involved in multiple signaling pathways including neuronal regulation and immunity. Accordingly, SD induced a decrease in 3-hydroxybutyric acid (3OBH, *p *= 0.0002) and an increase in thyroxine (T4, *p *< 0.0001) in plasma. The plasma anti-inflammatory cytokine IL-10 was downregulated while other ten inflammatory factors were upregulated.

**Conclusion:**

This study demonstrates that SD influences biochemical, physiological, cognitive variables, and the significantly changed variables may serve as candidates of SD markers. These findings may further our understanding of the detrimental consequence of sleep disturbance at multiple levels.

## 1 Introduction

In modern society, many people experience inadequate amount of sleep due to sleep disorders, irregular schedule, and excessive workload, etc. Insufficient sleep and circadian disruption cause numerous negative health outcomes, such as all-cause mortality, obesity, diabetes, cardiovascular disease, and impaired vigilance and cognition (1–4). It is crucial to systematically investigate the comprehensive influences of sleep insufficiency or deprivation on physiology and behaviour.

A cross-sectional study of white British adults aged 40–69 showed that among the subjects who slept shorter and longer than the normal 7–8 h duration, the levels of C-reactive protein (CRP, an inflammatory biomarker) and gamma glutamyltransferase (GGT, a liver function biomarker) were increased in those subjects, despite the modest changes in most blood biomarkers, suggesting the effects of SD on plasma biochemical variables (5). In rats, sleep deprivation resulted in decreased plasma concentration of free Mg^2+^ and Ca^2+^ electrolytes which may contribute to associated physiological problems (6).

Insufficient sleep led to altered gene expression and pathways associated with the circadian clock, sleep homeostasis, oxidative stress and metabolism, which are involved in chromatin modification, regulation of gene expression, macromolecular metabolism, and inflammatory, immune and stress responses (7). The association between sleep disturbance and inflammation has been reported in a number of studies (8–21). However, some of the reports remain inconsistent. For instance, it has been reported in some studies that SD can induce the inflammation factors IL-6 and TNFα (9–12), but a recent meta-analysis demonstrated that there were no significant changes in IL-6 and TNFα in sleep deprivation or sleep restriction (13). Therefore, the SD consequence on inflammation needs further validation.

circRNAs are a new class of RNA molecules characterized by their covalently closed circular structure, which regulate a diversity of cellular processes at the post-translational level, e.g., through acting as miRNA sponges, anchors for circRNA binding proteins (cRBPs), molecular scaffolds, and regulators of transcription and translation (22). circRNAs are abundantly present in brain and deregulation of circRNAs has been implicated in neurodegenerative, psychiatric, and neurodevelopmental disorders (23, 24). In mouse suprachiasmatic nucleus (SCN) and predicted *Cdr1as* circRNA as an essential regulatory molecule that impacts the light entrainment in the SCN through binding with *miR-7* (25). The study of the function and mechanism of circRNAs in sleep is very limited and systematic understanding of the circRNA roles in sleep regulation remain elusive.

Neurotransmitters coordinatively regulate sleep and wakefulness (26). At the molecular level, sleep disturbance causes alterations in the expression of a subset of metabolites, including some neurotransmitters or neurotransmitter receptors (27, 28). Benedict et al. reported that an acute SD increased the morning serum levels of neuron-specific enolase (NSE) and S100 calcium binding protein B (S-100) by approximately 20%, which reflects potential neuronal damage (29). However, the implication of only very few inflammation factors and neurotransmitters have been investigated in SD by far. In this study, we recruited 17 volunteers, conducted a 32.5-h SD experiment, and demonstrated the comprehensive effects of SD on physiology, cognition, and the plasma circRNA profiles which suggests its potential impacts on neural and immunity systems. Furthermore, we confirmed that SD can induce the altered expression of some neurotransmitters and inflammatory factors.

## 2 Methods

### 2.1 Participants

In total, 17 male volunteers (age, 32.0 ± 4.4 years; height, 173.1 ± 4.6 cm; weight, 70.5 ± 9.8 kg; data are the means ± SD) were recruited to participate in this study, and the experiment was carried out from Sep 2 to Oct 16 in 2021 at the SPAC Enter Space Science and Technology Institute, Shenzhen, China. We recruited only male volunteers was to avoid the potential gender difference in sleep and associated physiology and behaviour (30). General physical, psychological and routine blood tests were performed to exclude ineligible subjects. No medication, smoking, alcohol, or caffeinated drinks were allowed during the study.

### 2.2 Experimental procedures

**Figure 1:**
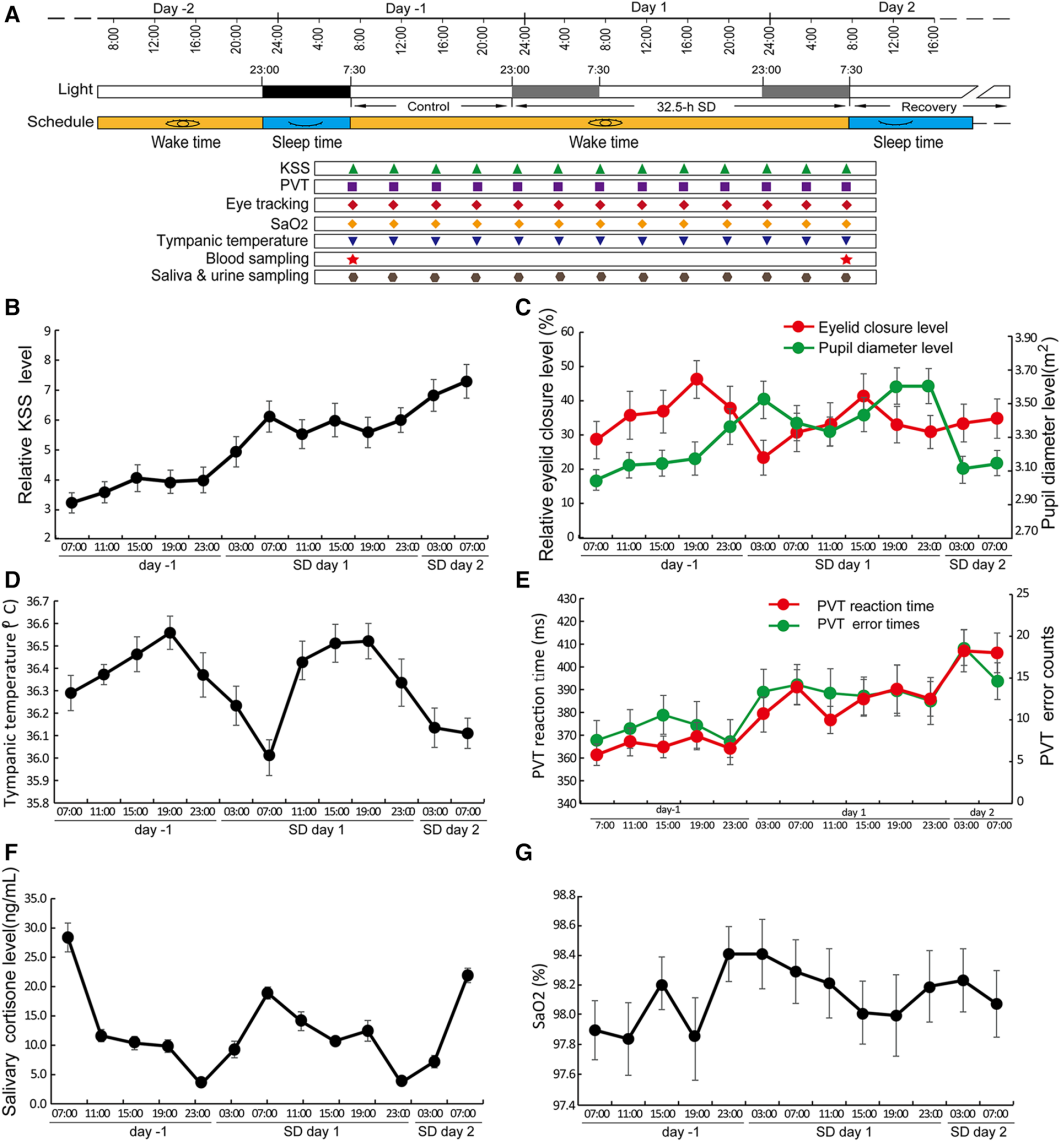
SD led to changes in a variety of circadian rhythms. (**A**) The schematic protocol of this study. The black block covering the time from -2 days to -1 day represents the sleep period, and the two grey blocks represent the same time blocks in the following two days but during which the subjects underwent SD. Different symbols in different colours denote the sampling or testing time points. (**B–F**) Circadian changes in KSS (**B**), eye tracking data (**C**), tympanic temperature (**D**), PVT RT and PVT error (**E**), salivary cortisone level (**F**), and SaO_2_ levels (**G**) Data are the mean ± SE. *n* = 17.

The experiment comprised a 2-d period prior to SD, 32.5 h of SD, and a 2-d recovery after SD. The participants arrived on -2 days prior to SD for adaptation to the environment. From day -2 to day -1, the sleep time was scheduled from 23:00 to 7:30; the subjects remained awake from 7:30 on day -1 to 7:30 and throughout day 2. The period from 7:30 to 23:00 on day -1 was considered the control period, and the period from 23:00 on day -1 to 7:30 on day 2 was considered the SD period. Participants were allowed to sleep or relax freely from 7:30 on day 2, which was the recovery period. The Karolinska Sleepiness Scale (KSS) questionnaire, Psychomotor Vigilance Task (PVT), and assessments of eyesight, tympanic temperature, SaO_2_, blood sampling and saliva sampling were performed every four h according to the schedule (±0.5 h). Blood was drawn after overnight fasting at approximately 7:00 (±0.5 h) for all subjects, and plasma samples were separated and stored at −80°C (Figure 1A). We measured the tympanic temperatures as a representative of core body temperature (CBT) with a Braun ThermoScan® PRO 6000 Ear Thermometer (Germany).

### 2.3 Questionnaire and cognitive tests

The KSS was used to measure subjective sleepiness, which scored from 1 (extremely alert) to 10 (extremely sleepy) (31). PVT tests were conducted used to measure the subjects’ alertness (32). In this study, the 3-min PVT-B was used, which comprises 30 visual stimuli; the random stimulus interval ranged from 2 s to 10 s (25). The two indicators of PVT alertness include reaction time (RT, ms) and errors (times). The PVT reaction time refers to the average response time, reflecting the performance speed. PVT errors refer to the number of errors (integers) out of the 30 stimuli reflecting performance accuracy (33). The eye tracking data were acquired using a Tobii Pro Spectrum (Tobii Pro, Sweden), including pupil diameter and eyelid closure. The screen was 60 cm from the eyes, the head was stabilized with a chin rest, and the sampling frequency was 150 Hz. Both the data of the pupil diameter and eyelid closure data were recorded. The relative eyelid closure PERCLOSE 80 (P80) was calculated according to the P80 standard (34).

### 2.4 Biochemical analysis of salivary cortisone

Salivette tubes (Sarstedt, Numbrecht, Germany; 51.1534.500) were used to collect salivary samples. The cortisone level in 100 µl saliva of each sample was measured with LCeMS/MS (TSQ Quantum Ultra), which comprises an ultimate 3,000 system and a Thermo Scientific TSQ Quantum Access Triple Stage Quadrupole Mass Spectrometer (Thermo Fisher, USA).

### 2.5 Microarray screening of differentially expressed circRNAs

Total RNAs were isolated using TRIzol reagent, and the purity and concentration were determined by NanoDrop ND1000 (Thermo Fisher Scientific). Subsequently, the total RNAs were digested with RNase R (1 U/μg, Cat# RNR07250, Epicenter) to enrich circular RNAs, which were then amplified, and transcribed into fluorescent cRNA using Arraystar Super RNA Labeling Kit (Cat# 074301, Arraystar) by following with the manufacturer's instruction. The labeled cirRNAs were subjected to hybridization onto Arraystar Human circRNA Array v2 which detects the expression of 13,617 circular RNAs (Aksomics, China). Agilent Feature Extraction software (version 11.0.1.1) was used to analyse acquired array images. A series of data processing including quantile normalization were performed by R software limma package. Differentially expressed circRNAs with statistical significance between four groups were identified by fold change cutoff or through Volcano Plot filtering (FC ≥2.0 and *p*-values ≤0.05). Heat map, Volcano plot and KEGG pathway analysis was performed using the OmicStudio tools at https://www.omicstudio.cn/tool.

### 2.6 Profiling of plasma neurotransmitters and inflammatory factors

Haemolysis occurred in the plasma samples from three volunteers; therefore, the samples of these three subjects were precluded for the assays of plasma neurotransmitters and inflammatory factors. The samples of the remaining 14 volunteers were subjected to metabolomic analysis of the expression profile of neurotransmitters and profiling of inflammation factors.

To measure the levels of plasma neurotransmitters, the samples were analysed using an LC-ESI-MS/MS system, and the analytical conditions were as follows: HPLC column, Waters ACQUITY UPLC HSS T3 C18 (100 mm × 2.1 mm i.d. 1.8 µm); solvent system, water with 0.1% formic acid (A), acetonitrile with 0.1% formic acid (B); gradient started at 5% B (0 min), increased to 95% B (0–8 min), 95% B (8–9.5 min), and finally ramped back to 5% B (9.6–12 min); flow rate, 0.35 ml/min; temperature, 40°C; and injection volume: 2 μl. Ultraperformance liquid chromatography (UPLC) (ExionLC™ AD) coupled with tandem mass spectrometry MS/MS (QTRAP® 6500+) was performed by Metware Biotechnology Co. Ltd. (China). The data were analysed with Analyst 1.6 software (AB Sciex).

Plasma inflammatory factors were profiled using an antibody array, the Human Inflammation Array Q3 (QAH-INF-3-2; Raybiotech, Inc., USA). Each sample was assayed four times. The fluorescence data were converted to concentration values with RayBio Q Analysersoftware (Raybiotech, Inc., USA). The detailed experiment was conducted by following the manufacturer's protocol (https://doc.raybiotech.com/pdf/Manual/QAH-INF-3.pdf). In the analysis of plasma neurotransmitters and inflammatory factors, changes with fold changes >1.2 or <0.83 (*p* < 0.05) were considered differentially expressed factors (DEFs) with statistical significance.

### 2.7 Statistics

GraphPad Prism (version 8.0.2) was used for statistical analysis. Mann-Whitney test (for non-normally distributed data) or Student's *t*-test (for normally distributed data) was used to analyse the differences between the control and SD groups in the variables. Data are means ± SD or SE as indicated. * *p* < 0.05, ** *p* < 0.01, *** *p* < 0.001.

## 3 Results

### 3.1 Disturbed circadian rhythms and decreased cognition caused by SD

Most of the tested variables showed circadian rhythms during the control and SD periods, including eyelid closure level, pupil diameter, PVT reaction time and error occurrence, sleepiness, core body temperature, and cortisone but not blood oxygen saturation (Figures 1B–G). The circadian rhythm of eyelid closure showed a 4-h phase delay compared to pupil diameter (Figure 1C). The PVT reaction time and error rate of PVT operation showed similar circadian patterns while the CBT and salivary cortisone showed roughly anti-phase patterns (Figures 1D–F).

**Figure 2:**
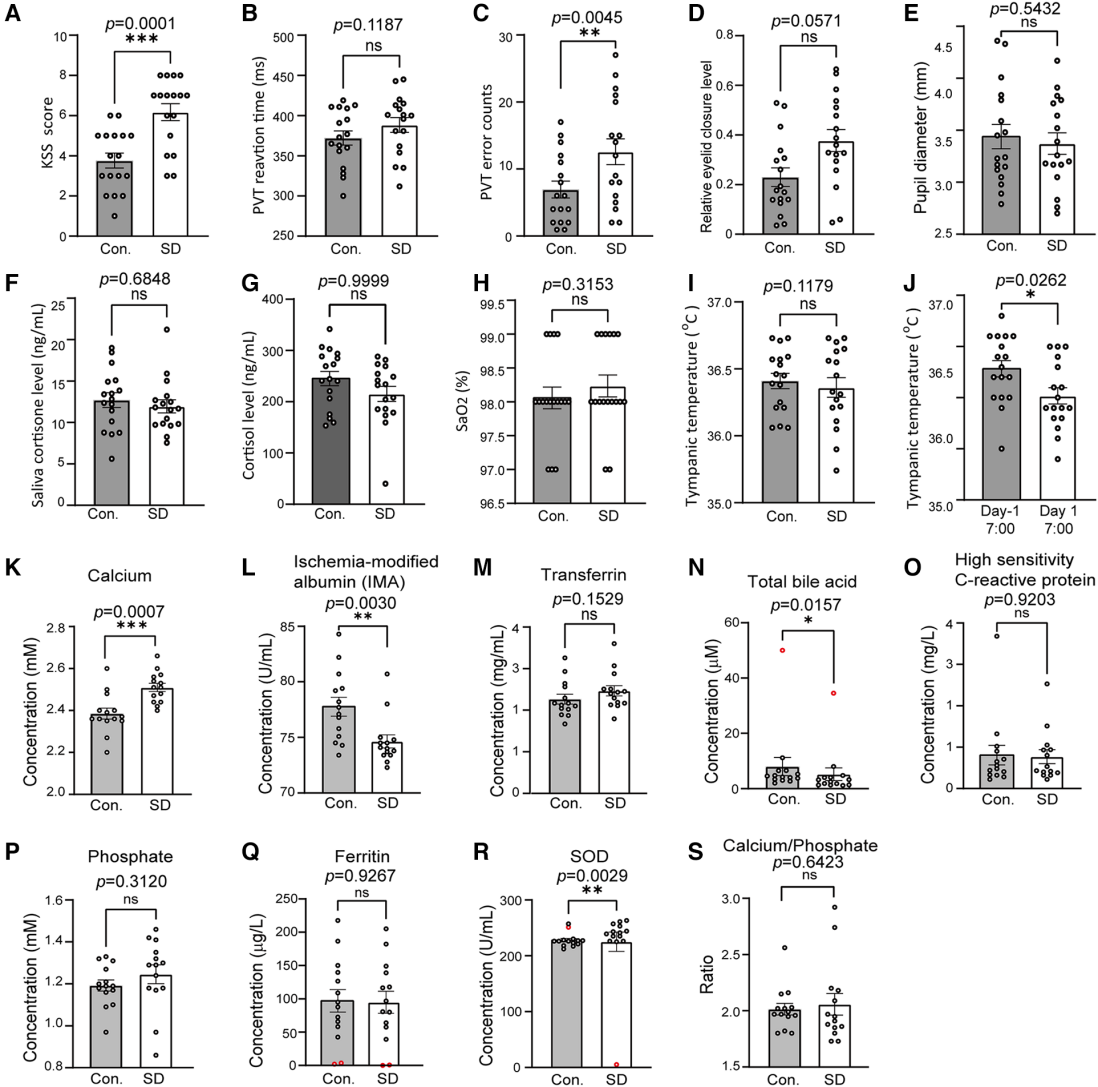
SD caused changes in a number of physiological, cognitive and biochemical variables. (**A–F**) The compared variables included the KSS score (**A**), PVT RT (**B**), PVT error (**C**), relative eyelid closure level (**D**), pupil diameter (**E**), salivary cortisone (**F**) and urinal cortisol (**G**), SaO2 (**H**), tympanic temperature (**I**), and tympanic trough values (**J**). The variables were compared between 7:30–23:00 on day -1 and 7:30–23:00 on day 1 (**A–I**). In J, the physiological, cognitive variables of tympanic trough temperature were compared between 7:30 on day -1 and 7:30 on day 1. *n* = 17. Panels A through I are the results of calcium (**K**), ischaemia-modified albumin (**L**), transferrin (**M**), total bile acid (**N**), high sensitivity C-reactive protein (**O**), phosphate (**P**), ferritin (**Q**), superoxide dismutase (**R**), and Ca/P ratios (**S**). *n* = 14, abnormal data were precluded for analysis (red circles). The data are shown as the mean ± SE. * *p* ≤ 0.05, ** *p* ≤ 0.01, *** *p* ≤ 0.001, n.s., nonsignificant.

Significant increases in KSS score and PVT error were observed (Figures 2A–D). There was no significant change in PVT reaction time, eyelid closure level, pupil diameter, salivary cortisone and urinal cortisol, SaO2 and the average level of tympanic temperature (Figures 2E–I). Cortisone is a metabolite of cortisol, the comparable levels of both cortisone and cortisol between control and SD groups suggest that no stress occurred during SD (Figures 2F,G). In contrast, the trough values of tympanic temperature at 7:00 were significantly decreased on day 1 compared to those on day -1 (Figure 2J), suggesting a decreased amplitude.

### 3.2 Altered plasma biochemical parameters due to sleep deprivation

The plasma samples of 14 volunteers were subjected to biochemical analysis and the results demonstrated that the level of calcium (*p *= 0.0007) increased after SD while IMA (*p *= 0.0030) and total bile acid TBA (*p *= 0.0157) decreased. The SOD level showed significant decrease (*p *= 0.0029) but the change was slight (Figures 2K–N). No significant changes were detected in the transferrin, high-sensitivity C-reactive protein, phosphate, ferritin, and plasma ratio of calcium/phosphate (Figures 2O–S).

### 3.3 Analysis of changed expression of circRNAs induced by sleep deprivation

**Figure 3:**
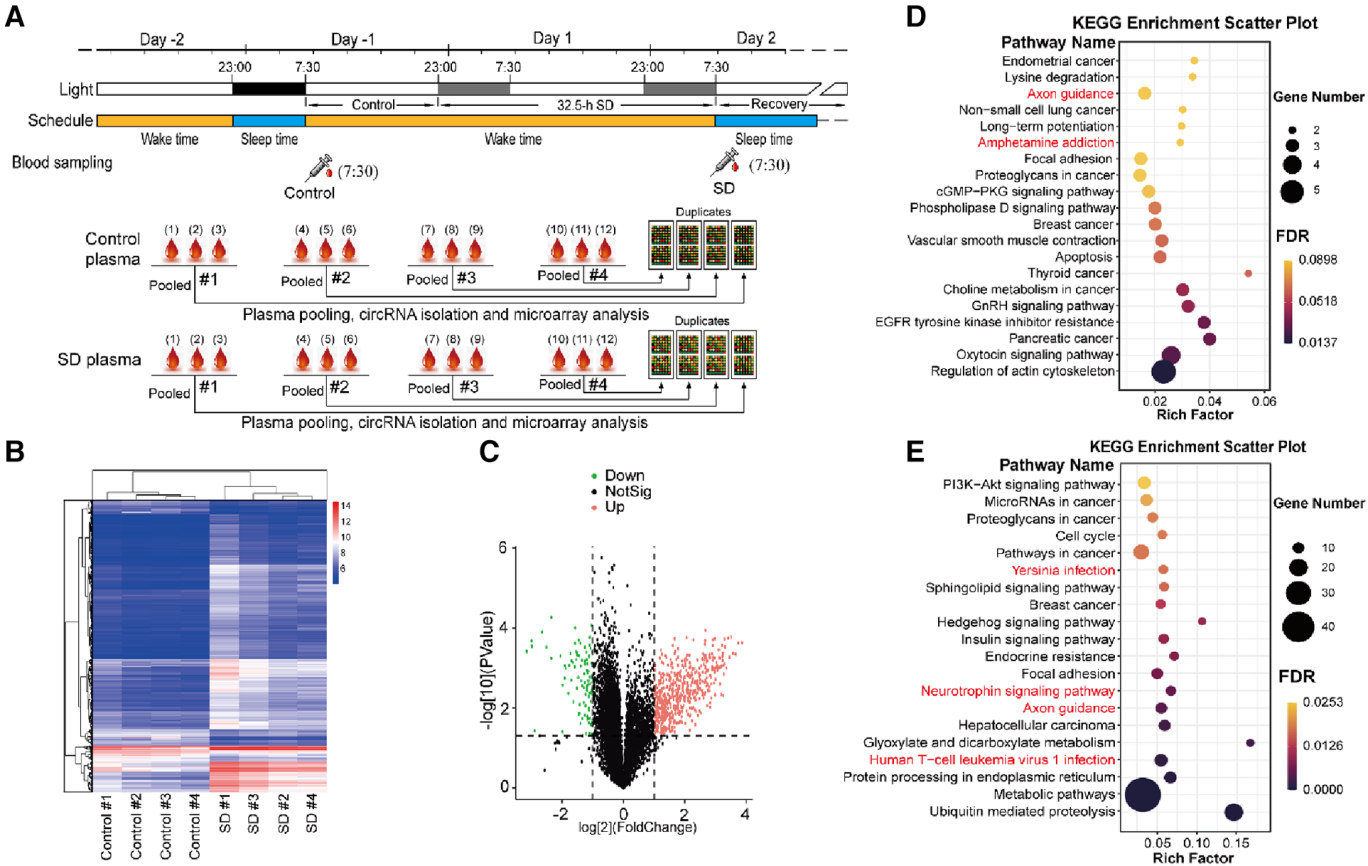
Analysis of differentially expressed plasma circRNAs. (**A**) The schematic diagram of plasma sampling and treatment for microarray analysis. Top: blood sampling was conducted around 7:30 on day -1 (control) and day 2 (SD), and plasma was isolated. Bottom: plasma samples from every 3 volunteers were pooled at random. Subsequently, total RNA was isolated and subjected to microarray analysis. (**B**) Heat map of the detected circRNA gene expression between control and sleep deprivation. The data of four groups of pooled plasma samples are present. Hierarchical Clustering was performed to show the distinguishable circRNAs expression pattern among samples. The heat map was constructed according to the microarray data. (**C**) Volcano plot that shows differentially expressed circRNA genes with statistical significance and fold change in the SD and control group. Significant genes were selected by fold change (>2 or <–0.5) and *p*-value (<0.05). (**D,E**) KEGG pathway analysis of upregulated (**D**) and downregulated circRNA genes (**E**) in SD compared to those in control (*p* < 0.05).

To investigate the molecular changes upon acute SD, in this study we conducted microarray to investigate the genes which are potentially associated with sleep deprivation. The plasma samples of twelve volunteers were pooled into four groups at random, and the total RNA was isolated and subjected for circRNA profiling using microarray. The samples from other volunteers were precluded as the quantity was insufficient (Figure 3A). The circRNA profiling results showed that SD induced differential expression of a set of circRNAs, which is well consistent between the four pooled groups (Figure 3B).

552 up-regulated and 94 down-regulated circRNAs induced by SD were identified (Figure 3C). The enrichment results from Kyoto Encyclopedia of Genes and Genomes (KEGG) analysis showed that many signaling pathways were altered due to SD including a number of important signaling pathways including PI3K-Akt signaling pathway, Hedgehog signaling pathway, cGMP-PKG signaling pathway and phospholipase D signaling pathway, many basic processed associated with cellular physiology including actin cytoskeleton, focal adhesion, cell cycle, protein processing in endoplasmic reticulum, and metabolic pathways including choline metabolism in cancer, insulin signaling pathway and metabolic pathways. Neuronal regulatory pathways were also identified, e.g., axon guidance, neurotrophin signaling pathway, ampheramine addiction. Moreover, many pathways involved in cancers and immunity were also identified, for instance, human T-cell leukemia virus 1 infection and hepatocelluar carcinoma (Figures 3D,E).

### 3.4 Changes in the profile of neurotransmitters and inflammatory factors caused by sleep deprivation

**Figure 4:**
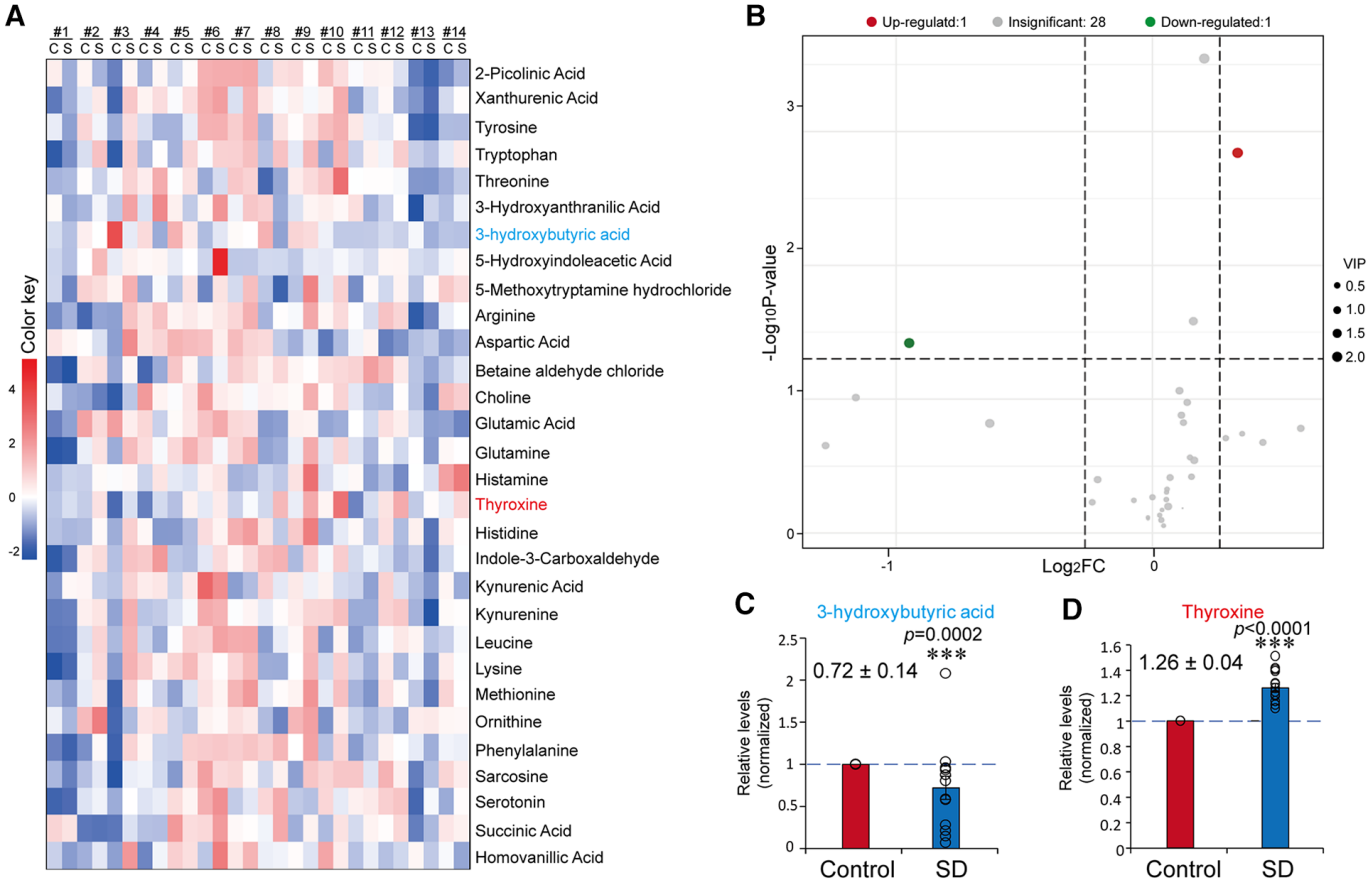
Sleep deprivation resulted in a changed profile of plasma neurotransmitters. (**A**) Heatmap of the results from targeted metabolomics analysis. Mean data of the concentration levels were used for drawing the heatmap. (**B**) Volcano plot of plasma neurotransmitters before and after SD. (**C,D**) Relative levels of 3OBH (**C**) and T4 (**D**) in the control and SD groups. The data were normalised to the control. Data are means ± SE. *n* = 14. Significance was determined by Student's *t*-test. * *p* ≤ 0.05, *** *p* ≤ 0.001.

As the circRNAs profiling results revealed the expression pathways associated with immunity and neuronal regulation, we next investigated the influences of SD on neurotransmitters and inflammatory factors. The plasma samples were subjected to targeted metabolomics analysis to examine the levels of 35 neurotransmitters or related metabolites. Among the neurotransmitters or related metabolites, 3-hydroxytyramine, serine, acetylcholine, 3-methoxytyramine and glycine were undetected in some of the samples; therefore, the data of these factors were precluded for further analysis. In those detected neurotransmitters, we found that the level of 3OHB was significantly decreased (0.72 ± 0.14, *p *= 0.0002), while the level of thyroxine increased (1.26 ± 0.06, *p *< 0.0001) (fold change >1.2 or <0.83) (Figures 4A–D).

**Figure 5:**
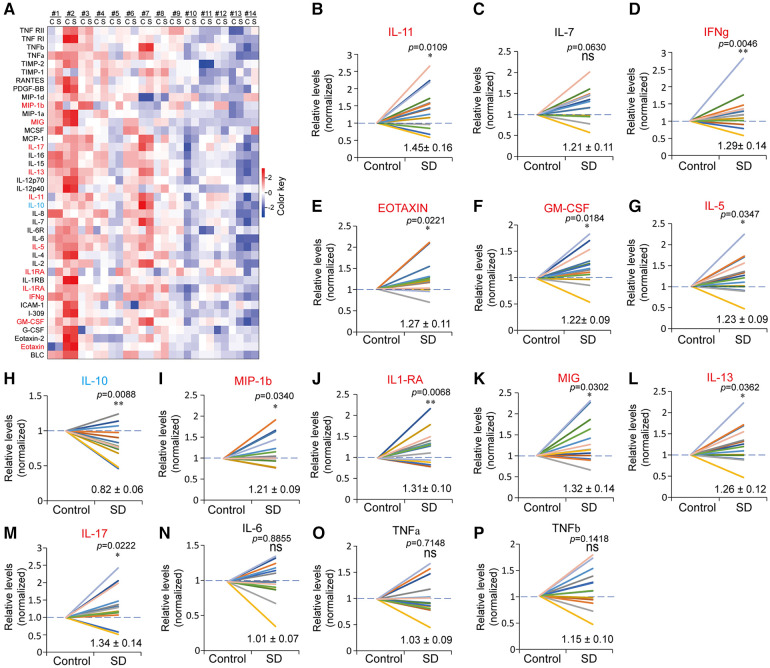
Altered profiles of plasma inflammatory factors due to sleep deprivation. (**A**) Heatmap of the plasma inflammatory factors in the control (**C**) and SD (**S**) groups detected by antibody microarray. The data were processed by plus one, log_2_ transformation and *z*-score normalization from raw data to draw the heatmap. (**B-P**) Relative levels of the indicated inflammatory factors in the control and SD groups. The data are shown as the mean ± SE. * *p* ≤ 0.05, ** *p* ≤ 0.01, *** *p* ≤ 0.001, n.s., nonsignificant.

To systematically probe the changes in plasma inflammatory factors induced by SD, we conducted an antibody microarray analysis that contained 40 antibodies in total. I-309 in the control of subject #4 and in the SD of subject #5 was undetected, which might be due to extremely low levels. Eleven inflammatory factors with significant changes were identified through antibody microarray analysis, among which IL-11, IFNg, EOTAXIN, GM-CSF, IL-5, MIP-1β, IL-1RA, MIG, IL-13, and IL-17 were upregulated (fold change >1.2), while IL-10 was downregulated (fold change <0.83) (Figures 5B–M). In contrast, IL-6, IL-7, TNFα and TNFβ showed no significant changes in this study (Figures 5N–P).

## 4 Discussion

In this study, the volunteers showed changes in a number of variables, including decreased alertness and increased sleepiness, which is consistent with previous reports (1, 35). Among these variables, the changes in circadian patterns of sleepiness, PVT parameters and salivary cortisone levels are consistent with previous reports (1, 36). In addition, the subjects showed a continuously decreased vigilance during SD. Together, these results validate the present experiments. The trough levels of tympanic temperature were significantly reduced despite its unchanged average level during SD (Figures 1D, 2J), suggesting an occurrence of disturbance in metabolism. The altered body temperature may contribute to decreased vigilance; in addition, the present results also suggest that tympanic temperature could be used to monitor the circadian rhythmicity of CBT.

Sleep restriction disturbs the homeostasis of bone metabolism (37–39). In this study, plasma calcium was significantly increased during SD (Figure 2K), which may be associated with bone absorption (40, 41). Furthermore, induced Ca^2+^ in SD plasma samples was observed in this study (Figure 2K), while it has been reported that in rats sleep deprivation resulted in decreased plasma concentration of Ca^2+^ (6). This inconsistency may be attributed to species difference or different SD durations. Previously we found that sleep deprivation plus non-24-h routine resulted in significant upregulation of TBA (42), while here we found a significant decrease in plasma TBA (Figure 2N). Despite of the different changes, these findings together suggest a potential detrimental influence of SD on the functions of liver and hepatic duct.

circRNAs regulate a diversity of cellular processes at the post-translational level and they play important roles in the neural development and progression of neurological disorders (43). In this study, we identified the alteration of circRNAs implicated the amphetamine addiction pathway (Figure 3). SD can potentiate the mesolimbic dopaminergic availability and function, which mimics the neuropharmacological effects of amphetamine (44). In addition, the pathways regulating neurotrophins and axon guidance were also identified (Figures 3D,E). Neurotrophins, e.g., brain-derived neurotrophic factor (BDNF), are prominent regulators of neuronal survival, growth and differentiation during development. BDNF plays an important role in the pathophysiology of many neurodegenerative disorders, depression, anxiety and other psychiatric disorders (45). Sleep-dependent synaptic plasticity is crucial for optimal cognition, and the axon guidance proteins control structural plasticity of synaptic connections. Disrupted axon guidance underlies some neurological disorders which are characterised by structural changes in neuronal connections (46). These facts suggest that SD may cause deleterious effects on neural and cognitive functions.

Neurotransmitters coordinatively regulate sleep and wakefulness (26). For instance, γ - aminobutyric acid (GABA) inhibits the firing of cells associated with wakefulness (47). As a neurotransmitter, adenosine has been reported to be induced in an SD experiment (48). By using the targeted LC/MS metabolomics method, we found that the levels of circulatory 3OBH and thyroid hormone were altered due to sleep loss. 3OHB, serves as an energy source of acetyl coenzyme A for maintaining neural function when the plasma glucose level is reduced (49). At the molecular level, 3OHB regulates neuronal metabolism by increasing mitochondrial respiration, which enhances the expression of BDNF in cultured cerebral cortical neurons (50). In addition, 3OHB is an endogenous and specific inhibitor of class I histone deacetylases (HDACs), which protect neurons against excitotoxicity and oxidative stress and inhibit inflammatory activation in Alzheimer's disease (51, 52).

Thyroid hormone thyroxine is the major thyroid hormone that controls neural development and functions of the central nervous system (53, 54). It has been proposed that T4 is positively associated with acetylcholine and cognition (54, 55). In this study, we found that T4 was significantly but not dramatically induced by SD (Figure 4D). Supportively, elevated thyroxine was also found in other studies involving partial sleep restriction and SD (56–58). Considering the decreased vigilance, the increased T4 may represent a protective response. Furthermore, the induction of thyroxine may account for the higher plasma calcium (59). However, inconsistent changes in thyroxine have also been reported. For instance, T4 reduction was observed mainly in female participants in a sleep restriction study (60). In rats, the levels of free thyroxine were decreased in two different SD experiments (61, 62). Moreover, significant increases in tryptophan and serotonin wakefulness have been demonstrated during a continuous 24-h period (27). In contrast, in this work, no significant changes in either tryptophan or serotonin were observed (Figures 4A,B). These inconsistences suggest that the effects of SD on these factors remains further validation.

Sleep disturbances lead to various consequences on immunity, e.g., increased risks of infection and cancer, exacerbation of autoimmune diseases, neurodegenerative diseases and metabolic and vascular diseases (15–19). In different SD experiments, the induction of IL-6 and TNFα has been reported (9–12). Furthermore, changes in the levels of other inflammatory factors were observed, for instance, the activation of toll-like receptor-4, STAT1, STAT3, STAT5, TNF-α receptor R1, and TLR-4, and the reduction of IGF-I (11, 14, 20). At the mRNA level, a 25-h sleep deprivation induced the expression of whole-blood of *TNF-*α and its receptors *R1* and *TLR4*, while *IL-6* remained unchanged (9). The nocturnal increase in IL-6 occurred in stage 1–2 sleep and rapid eye movement sleep, and partial sleep deprivation delayed the increase in the plasma level of IL-6 (21).

Nonetheless, a recent meta-analysis excluded the association between IF-6 and TNFα and sleep deprivation or sleep restriction (13). Consistently, we systematically measured the protein levels of 40 using antibody microarray and found that there was no significant change in plasma IL-6, TNFα and TNFβ between the control and SD groups. Instead, decreased plasma levels of IL-10 and increased levels of ten factors were found in this study, which play roles in promoting tissue inflammation (Figure 5). IL-10 is an anti-inflammatory cytokine that limits or suppresses immune responses (63). In contrast, the ten factors, which were induced by SD, function to promote immune responses or inflammation through different pathways. In addition, the decrease in 3OBH supports the occurrence of inflammation, as it is an inflammation inhibitor. The converse alterations of IL-10 and the ten inflammation factors suggest a disturbance of the balance between proinflammatory and anti-inflammatory effects (64). These data suggest that sleep insufficiency may cause immunity disturbance. Interestingly, a recent study revealed that in mice a 4-days long SD induced severe inflammation through elevating the prostaglandin D_2_ efflux across the blood-brain-barrier (65). This mechanism may also account for the upregulation of inflammation factors caused by SD in human.

## 5 Conclusions

In this study we demonstrated that sleep deprivation can cause extensive physiological, biochemical and cognitive changes. The neurotransmitters and inflammatory factors identified in this study may serve as potential biomarkers for sleep deprivation. The findings in the present study may provide new insights into the adverse consequence of sleep deprivation and the regulation of human sleep.

## 6 Limitations

The volunteers lived in a well-controlled indoor environment in the institute, due to the limited room, high organization workload and expense for the tests and measurements, we recruited only 17 volunteers in this study. In the future, some of the results may be necessary to be validated in a larger size of samples. In addition, we only drew the blood samples at two time points prior to and after SD in this study. If the blood samples had been drawn at more time points, the changes in the rhythmicity of plasma neurotransmitters and inflammatory factors could be analysed, which may provide more helpful information in understanding the SD effects.

## Data Availability

The datasets are available upon reasonable request from the corresponding author.
